# Clinical usefulness of iQ200/iChem Velocity workstation for screening of urine culture

**DOI:** 10.1186/s12879-019-4141-x

**Published:** 2019-06-17

**Authors:** Jong-Mi Lee, Doo-Jin Baek, Kang Gyun Park, Eunhee Han, Yeon-Joon Park

**Affiliations:** Department of Laboratory Medicine, College of Medicine, Seoul St. Mary’s Hospital, Catholic University of Korea, Banpodaero 222, Seocho-gu, Seoul, 06591 South Korea

**Keywords:** Urinary tract infection, Urine culture, Combination, WBC and all small particles

## Abstract

**Background:**

Clinical microbiology laboratories are asked to process large numbers of urine specimens for culture, but only 20–40% of them are positive. Therefore, a rapid, reliable screening method is necessary to speed up the reporting of a negative result. In this study, we evaluated the iQ200/iChem workstation, which is a combination of digital imaging software and a strip reader to predict negative urine culture.

**Method:**

A total of 1942 urine specimens were processed through both culture and iQ200/ iChem workstation. We analyzed the performance using two definition of positive urine culture; one or two potential uropathogens at a concentration of ≥10^5^ CFU/ml and ≥ 10^4^ CFU/ml. We assessed combinations of parameters (ASP; all small particles, WBC; leukocyte, BACT; bcteria, LE; leukocyte esterase) applying various cut-offs which can achieve the negative predictive value (NPV) ≥97% and culture reduction rate ≥ 50%.

**Results:**

The culture positive rate was 12.8 and 18.4% applying the criteria of ≥10^5^ CFU/ml and ≥ 10^4^ CFU/ml, respectively. The area under the curve (AUC) of each parameter for ≥10^5^ CFU/ml / ≥10^4^ CFU/ml bacteriuria was 795 /0.719 for WBC, 0.722 / 0.701 for ASP and 0.740 /0.704 for bacteria. Therefore, we investigated the combination of the parameters. With the fixed parameter of BACT≥1/HPF and positive LE, the combinations of WBC ≥ 4/HPF and ASP ≥8500/μl or WBC ≥ 6/HPF and ASP≥5500/μl showed good performance for detecting ≥10^5^ CFU/ml uropathogen. The ranges of sensitivity, specificity, negative predictive value and culture reduction rate were 91.5–92.3%, 49.8–52.6%, 97.7–97.9% and 50.4–53.0%, respectively. However, none of the combined setting yielded acceptable range of NPV for detecting ≥10^4^ CFU/ml uropathogen (NPV 92.9–94.9%). *Enterococcus* spp. was the most common uropathogen causing the false negative results (55.7%), and also the main pathogen among the positive culture of 10^4-5^ CFU/ml bacteriuria (45%).

**Conclusions:**

iQ200/iChem workstation was excellent in detection of ≥10^5^ CFU/ml uropathogen, but unsatisfactory in detection of 10^4–5^ CFU/ml uropathogen and *Enterococcus* spp. It can be useful for screening of urine specimens to reduce bacterial culture. However, notice from clinician will be necessary for specimens from the patients with high risk for UTI, such as pregnant woman, infant, elderly or immune compromised patients.

**Electronic supplementary material:**

The online version of this article (10.1186/s12879-019-4141-x) contains supplementary material, which is available to authorized users.

## Background

Urinary tract infection (UTI) is the most common infection in both hospitalized and community patients [1]. The indications for urine culture are 1) when signs or symptoms suggest a UTI is present 2) in patients who cannot provide clinical history (intubated, demented) and 3) have sepsis without another source to explain it [2]. However, clinical microbiology laboratories are asked to process large numbers of urine specimens for culture, often in the absence of clear indications, giving negative results in 60–80% of specimens [3]. Therefore, a screening method that identify negative urine culture samples and exclude them from further culture procedures could reduce the overall turnaround time, workload, and costs. However, high sensitivity and negative predictive value (NPV) should be prerequisites to prevent the true UTI-positive urine specimens from not being cultured. Many screening methods have been introduced and evaluated for a long time. Gram staining is rapid and economical, but labor intensive and trained technologists are required. Moreover, it is not sensitive, and requires the concentration of bacteria more than 10^5^ CFU/ml in the urine [4]. Dipstick tests for leukocyte esterase and nitrite are rapid, inexpensive and simple but insensitive [5]. Active researches have continued to find a reliable and cost-effective automated urine screening system to exclude urine samples from unnecessary culture processes, but no method has yet proved truly reliable. The iQ200/iChem workstation (Iris Diagnostics, Chatsworth, CA, USA) is combination of iQ200 and a strip reader, iChem Velocity. The main difference of iQ200/iChem workstation from the flow cytometer is that the urine content is analyzed by assessment of digital images of the particles passing in the front of a microscope objective. This system has recently added a new parameter, the detection of “all small particles” (ASP), to improve the sensitivity of the assay. Six parameters from the two instruments (iQ200 and iChem Velocity) are available; ASP (all small particles), WBC (white blood cells, leukocyte), BACT (bacteria), BYST (yeast), LE (leukocyte esterase) and NT (nitrite), but the best combination of them is still uncertain. In this study, we investigated the combination of parameters using various cut-off values to maximize negative predictive value (NPV) and culture reduction rate of iQ200/iChem workstation to detect UTI in the patient population of a tertiary hospital in Korea.

## Methods

A total of 2530 urine samples submitted for bacterial culture from March to May 2017 to the clinical microbiology laboratory of the Seoul St. Mary’s hospital were enrolled without any selection. Of them, 1942 samples which had sufficient volume (> 4 ml) were included. They were from outpatients or hospitalized patients, and mostly midstream urine (1618, 83.3%), followed by catheterized urine (236, 12.2%). Samples were collected in sterile containers without preservatives and transported to the laboratory within 1 h after collection and was stored at 4 °C for maximum of 16 h before being processed. Each sample was divided into two aliquots; one was used for iQ200/iChem workstation and the other was used for routine urine culture. The order on processing and urine portion for the two tests were randomly determined. The parameters reported by the instrument include ASP, WBC, BACT, BYST, LE and NT. The indicators measured by the iQ200 module were ASP, WBC, BACT, and BYST and those by iChem Velocity were NT and LE. (The raw dataset used for this analysis can be found in Additional file 1). 

This study was approved by the institutional review board (IRB) of Seoul St. Mary’s Hospital. The IRB waived the requirement to obtain informed consent from participants, because we used de-identified left over samples obtained from routine examination.

Urine culture was performed using 1 μl disposable loop by delivering and spreading well-mixed urine onto the blood agar plate, and MacConkey agar plate. When overgrowth of Gram-negative bacteria was observed, colistin-nalidixic acid-supplemented Columbia blood agar was additionally used. The plates were incubated under aerobic condition at 37 °C for 16 to 24 h. In this study, the positive urine culture result was defined as the growth of one or two potential uropathogens with a concentration of ≥10^5^ CFU/ml respectively. Additional analysis was also performed based on cut-off value of ≥10^4^ CFU/ml for comparison with the previous studies, that investigated iQ200/iChem workstation [6, 7]. Potential uropathogens were defined as members of the family *Enterobacteriaceae*, *Enterococcus* spp., *Streptococcus agalactiae*, *Pseudomonas species, Acinetobacter baumannii complex, Candida* spp.*, Staphylococcus aureus, Staphylococcus saprophyticus*, *Aerococcus urinae* [8]. Grown colonies were identified by Vitek-2 system or Vitek MS (bioMerieux). Gram staining was also performed using 10 μl of well-mixed, unspun urine samples. The Gram stain slides were examined by a trained technician, and the presence of WBC were determined as ≥1/LPF [9]. First, we assessed the parameters using the manufacture-suggested abnormal thresholds for ASP (≥7500/μl), WBC (≥6/HPF), BYST (≥1/HPF) and BACT (≥1/HPF) and positive results for LE and NT (Customer Guide to iQ®200 Series Formed Particle Settings). Afterwards, we analyzed the results using the combinations of parameters applying various cut-off values which can achieve the NPV ≥ 97% and culture reduction rate ≥ 50%. Receiver operating characteristics (ROC) curves were made using MedCalc (v.16.4.3), Statistical analysis was performed using SPSS software version 24.0 (SPSS Korea, Seoul, Korea). A two-tailed *P* value < 0.05 was considered statistically significant.

## Results

During the study period 1942 samples were tested by iQ200/iChem workstation and cultured as routine process. The median age of the study population was 58 years old (range 1 days to 96 years), and 953 (49%) of them were male. Two hundred forty-eight samples (14.5%) revealed growth of ≥10^5^ CFU/ml of potential uropathogen, and the most common pathogen was *Escherichia coli* (33.5%) and was followed by *Candida* spp. (27.0%), *Enterococcus* spp. (20.6%).

One hundred nine samples (6.4%) showed growth of 10^4–5^ CFU/ml of potential uropathogen and the most common pathogen was *Enterococcus* spp. (33.9%) and was followed by *E. coli* (26.6%), *Candida* spp. (19.3%) (Table 1.). Two hundred thirty-three samples showing growth of non-uropathogens (*N* = 109) or growth of more than 3 isolates (*N* = 124) were considered as negative. According to the patient group, the prevalence of positive urine cultures (≥10^4^ CFU/ml) was highest in women older than 60 years old (157/444, 35.3%), followed by women between 18 and 60 years old (84/459, 18.3%), men older than 60 years old (72/423, 17.0%), men younger than 18 years old (15/107, 14.0%) and lowest in men between 18 and 60 years old (24/423, 5.7%). According to the type of the urine samples, catheterized urine showed higher positive rate (84/236, 35.6%), than that of midstream urine (273/1618, 16.9%) (Table 2). For detecting WBCs among the culture-positive specimens (≥10^5^ CFU/ml / ≥10^4^ CFU/ml bacteriuria), WBC was observed in 49.8%/41.2% by Gram stain, while iQ200 showed ≥6 WBC/HPF in 88.5%/63.1% of the culture-positive samples (*P* < 0.001 for both ≥10^5^ CFU/ml / ≥10^4^ CFU/ml bacteriuria) (Table 3).

**Table 1:** Demographic of the culture results

Sample Characteristics and Culture results	No. of samples^a^ (%)	
Growth behavior of the specimens		
Growth of non-uropathogen, multiple bacterial morphotypes	233 (12.0)	
No growth - culture negative	1225 (63.1)	
10^3^~10^4^ CFU/mL- culture negative	127 (6.5)	
10^4^~10^5^ CFU/mL- culture positive	109 (5.6)	
≥10^5^ CFU/mL- culture positive	248 (12.8)	
Species distribution culture positive samples.	**10** ^**4–5**^ **CFU/ml total/single (%)** ^**b**^	**≥10**^**5**^ **CFU/ml** **total/single (%)** ^**b**^
*Escherichia coli*	29/25 (26.6)	83/83 (33.5)
*Enterococcus* spp.	37/36 (33.9)	51/41 (20.6)
*Candida* spp.	21/21 (19.3)	67/67 (27.0)
*Klebsiella pneumoniae*	4/2 (3.7)	17/10 (6.9)
*Enterobacter* spp.	2/0 (1.8)	9/4 (3.6)
other *Enterobacteriaceae*	2/0 (1.8)	9/1 (3.6)
*Citrobacter* spp..	2/0 (1.8)	7/2 (2.8)
*Proteus mirabilis*	2/1 (1.8)	5/4 (2.0)
*Acinetobacter baumannii* complex	0/0 (0)	6/5 (2.4)
*Pseudomonas aeruginosa*	1/1 (0.9)	4/3 (1.6)
*Staphylococcus aureus*	3/1 (2.8)	2/2 (0.1)
*Streptococcus agalactiae.*	3/2 (2.8)	1/0 (0.4)
*Morganella morganii*	1/0 (0.9)	1/0 (0.4)
*Aerococcus urinae*	1/0 (0.9)	0/0 (0)
*Serratia marcescens*	0/0 (0)	1/0 (0.4)
*Pasteurella aerogenes*	0/0 (0)	1/0 (0.4)
*Streptococcus uberis*	0/0 (0)	1/0 (0.4)

^a^ The total number of samples was 1942

^b^ Total counts of the pathogen identified including mixed growth and single growth / counts of the single pathogen growth (percentage of total counts of each species divided by the number of 10^4–5^ CFU/ml - culture positive (109) or ≥ 10^5^ CFU/mL- culture positive(248))

**Table 2:** Culture results according to the gender, age, specimen types

Culture results	No. of samples (%)	10^4^–10^5^ CFU/mL (%)	> 10^5^ CFU/mL (%)
Gender/Age			
Women older than 60 years old	444 (22.9)	41 (9.2)	116 (26.1)
Women between 18 and 60 years old	459 (23.6)	30 (6.5)	54 (11.8)
Women younger than 18 years old	86 (4.4)	4 (4.6)	1 (1.2)
Men older than 60 years old	423 (21.8)	19 (4.5)	53 (12.5)
Men between 18 and 60 years old	423 (21.8)	5 (1.2)	19 (4.5)
Men younger than 18 years old	107 (5.5)	10 (9.3)	5 (4.7)
Specimen types			
Midstream urine	1618 (83.3)	89 (5.5)	184 (11.3)
Catheterized urine	236 (12.2)	20 (8.5)	64 (27.1)
Unknown	88 (4.5)	0 (0)	0 (0)

**Table 3:** Comparison of WBC detection by Gram stain and iQ200

(Gram stain) / (iQ200)	> 10^5^ CFU/mL (%) (*n* = 248)	> 10^4^ CFU/mL (%) (*n* = 342)
(+)/(+)	97 (40.9)	113 (33.0)
(+)/(−)	21 (8.9)	28 (8.2)
(−)/(+)	89 (37.6)	103 (30.1)
(−)/(−)	30 (12.7)	98 (28.7)

Gram stain results were available in 1810 samples

The positive WBC result in gram stain is defined as ≥1/LPF; The positive WBC result in iQ200 is defined as ≥6/HPF

**Fig. 1:**
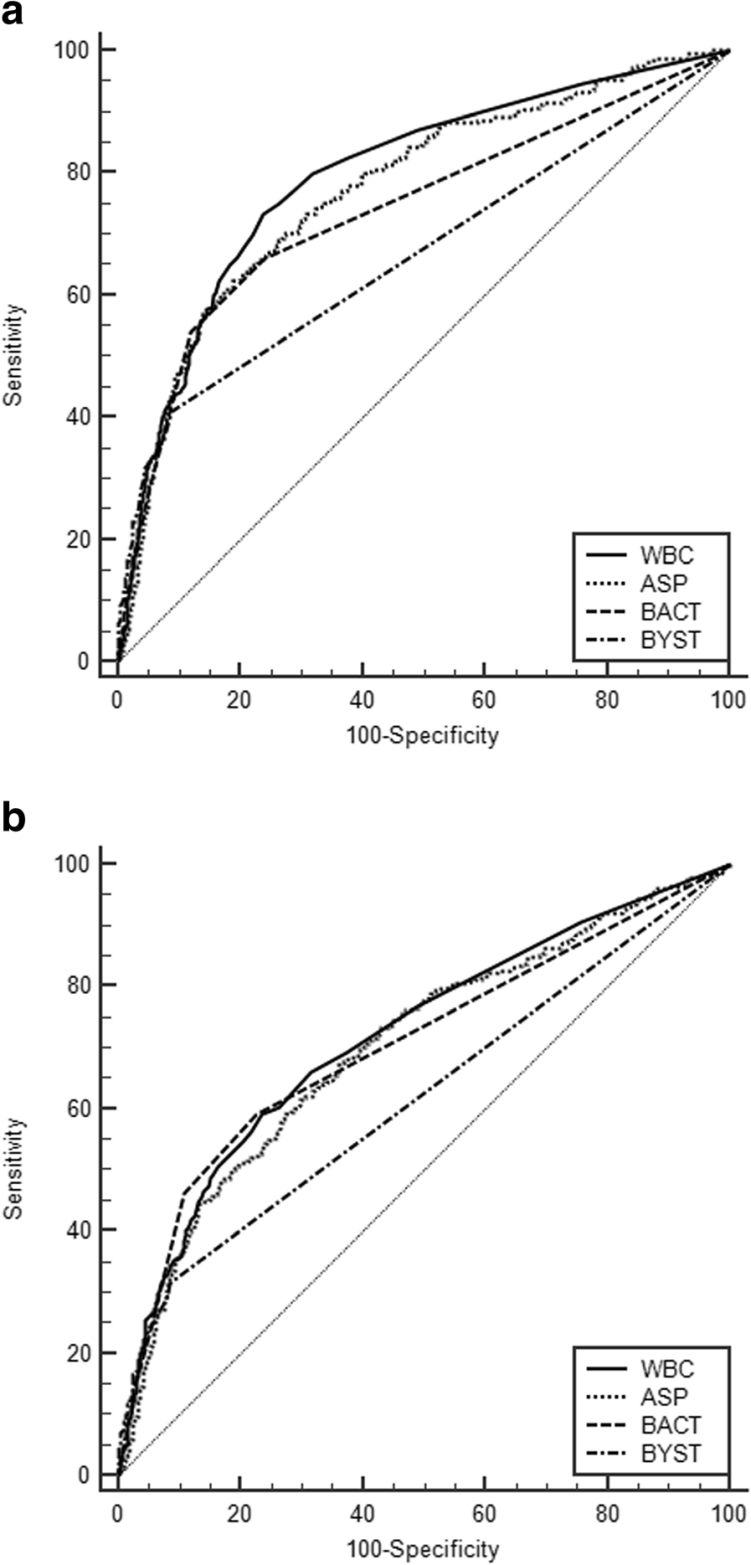
Receiver operating characteristic curves of the 4 parameters. **a** 10^5^ CFU/ml: positive urine culture, AUC of the parameter (95% confidential interval); WBC 0.795 (0.777–0.813), ASP 0.722 (0.753–0.791), BACT 0.740 (0.720–0.759), BYST 0.667 (0.646–0.688) (**b**) 10^4^ CFU/ml: positive urine culture, AUC of the parameter (95% confidential interval); WBC 0.719 (0.698–0.739), ASP 0.701 (0.680–0.721), BACT 0.704 (0.683–0.724), BYST 0.619 (0.597–0.641)

**Fig. 2:**
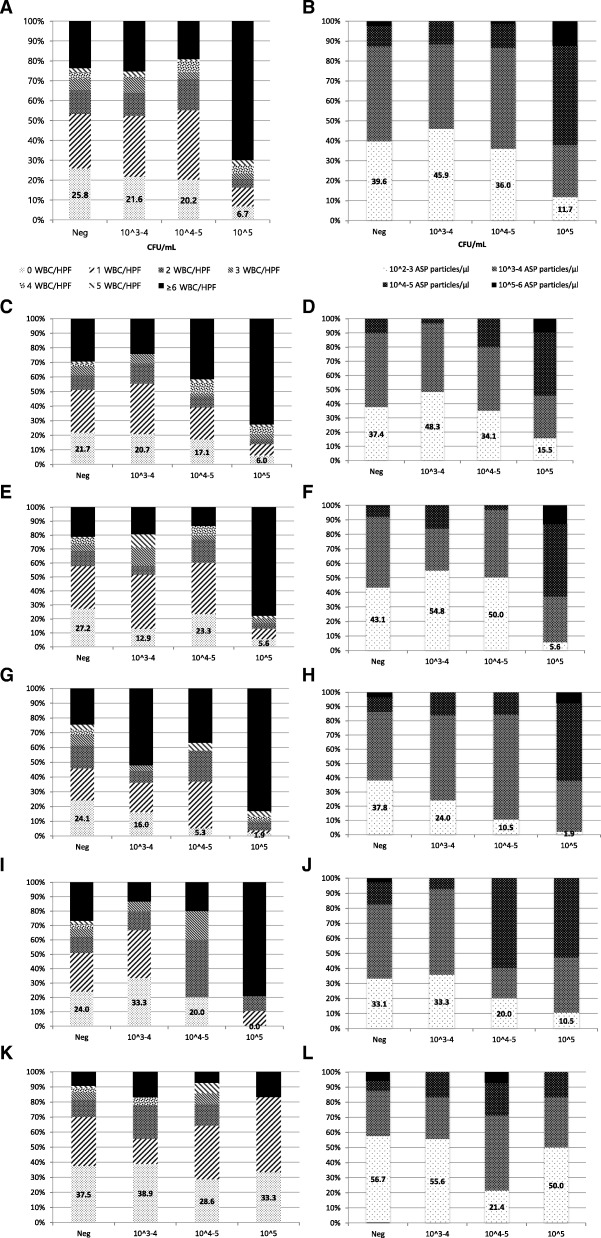
Stacked bar charts of growth uropathogen versus WBC and ASP. Urine samples from total study population (**a**,bb), women older than 60 years old (**c**,**d**), women between 18 and 60 years old (E,F), men older than 60 years old (G,H), men between 18 and 60 years old (I, J), patients younger than 18 years old (K,L)

Among the six parameters, as BYST and NT were not effective in detecting bacteriuria, we investigated the performance of the remaining four parameters by receiver operating characteristic curve (ROC) analysis (Fig. 1). For ≥10^5^ CFU/ml and ≥ 10^4^ CFU/ml of bacteriuria, WBC showed highest AUC (0.795/0.719), followed by ASP, BACT and BYST (0.722/0.701, 0.740/0.704 and 0.667/0.619, respectively). Moreover, even if the cut-off of WBC ≥ 1/HPF was applied, 20.2% of urine culture positive samples containing 10^4–5^ CFU/ml and 6.7% of urine culture positive samples containing 10^5^ CFU/ml will be regarded as falsely negative (Fig. 2-a). Likewise, with the cut-off value of ASP≥1000/μl, as many as 36.0% of urine culture positive samples containing 10^4–5^ CFU/ml, and 11.7% of urine culture positive samples containing 10^5^ CFU/ml will be regarded as falsely negative (Fig. 2-b). According to the patient group, the adult women group showed similar pattern with the total population (Fig. 2-c,f), while the adult men groups showed the higher sensitivity than total population in both WBC and ASP (Fig. 2-g,h). Notably, the both parameters showed poor performance in patients younger than 18 years old (Fig. 2-K,L).

**Fig. 3:**
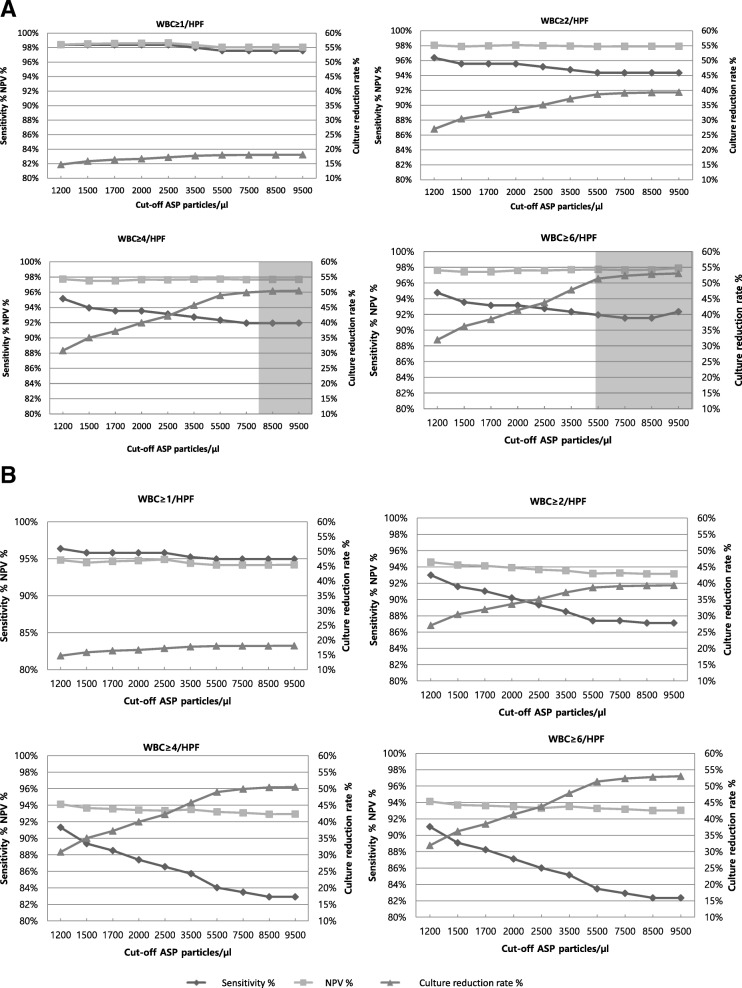
Relationship of sensitivity, NPV and culture reduction rate according to the WBC and ASP cut-off values. The grey shadow areas indicate the cut-off value combinations showing acceptable range of NPV (> 97%) and culture reduction rate (> 50%). Six combinations that satisfy the acceptable range for detecting uropathogen of 10^5^ CFU/ml are shown in the presented charts. However, none of the combinations gives acceptable results for detection of 10^4^ CFU/ml. The raw data of the charts are presented in the Additional file 2: Table S1

Because the individual parameters were not effective, we assessed the performance of the combination of WBC and ASP with a fixed parameters of BACT≥1/HPF and positive LE (Fig. 3, Supplementary Table S-1,2). For 10^5^ CFU/ml bacteriuria, NPV and sensitivity were high (> 97 and > 91%) regardless of WBC or ASP cut-off value. However, to attain the culture reduction rate of ≥50%, we chose WBC ≥4/HPF and WBC ≥6/HPF. When WBC ≥4/HPF was applied, the acceptable NPV (97.7%) and culture reduction rate (50.4–50.5%) were obtained at ASP≥8500/μl. Likewise, when WBC ≥6/HPF was applied, the acceptable NPV (97.7–97.9%) and culture reduction rate (51.4–53.0%) were obtained at ASP≥5500/μl (Fig. 3c-d). These combinations include the abnormal thresholds suggested by manufacturer (WBC ≥ 6/HPF, ASP≥7500/μl, BACT≥1/HPF and positive LE). By using it, the sensitivity, specificity, positive predictive value (PPV), NPV and culture reduction rate were 91.5, 51.5, 21.7, 97.7 and 52.3%, respectively. False negative results were observed in 21 cases, which include 8 *Enterococcus* spp., 5 *Escherichia coli*, 3 *Enterobacter* spp., 3. *Candida* spp., and so on.

In detection of 10^4^ CFU/ml bacteriuria, none of the combined settings could achieve the acceptable NPV or culture reduction rate. By applying the manufacturer’s suggested thresholds, the sensitivity, specificity, PPV, NPV and culture reduction rate were 87.4, 39.3, 24.5, 93.3 and 39.1%. False negative results were observed in 61 cases, which include 34 Enterococcus spp., 14 Escherichia coli, 4 Enterobacter spp., 4. Candida spp., and so on.

## Discussion

The main objective of this study was to evaluate the automated urine screening.

system to reduce the number of specimens to be cultured without excluding the true UTI.

specimens. As individual parameter showed low sensitivity, we assessed the combination of parameters. With the fixed parameter of BACT≥1/HPF and positive LE, we found that either the combination of WBC ≥4/HPF+ ASP ≥8500/μl or WBC ≥6/HPF + ASP ≥5500/μl, could achieve the NPV ≥97% and culture reduction rate of ≥50% in detection of 10^5^ CFU/ml bacteriuria, and the latter includes the abnormal thresholds recommended by the manufacturer.

Previous investigators reported various results in evaluation of iQ200: Parta et al. [7], reported 94.2% NPV and culture saving rate of 48.9% at the combined settings of ASP ≥10,000/μl, WBC ≥6/HPF, and few or more bacteria or yeast, at culture positive rate of 25.6%. In the study of Sturenburg et al. [6], at a given sensitivity of 95%, the culture saving rate was 35% and specificity was 61% under the various combinations of ASP+WBC + BACT+NT + LE. However, it is difficult to directly compare the study results, because of the differences in the study designs and patient populations that may affect the distribution of uropathogens (species and concentrations). Furthermore, several investigators have improved performance through reclassification of the captured image by trained technologists [10–13]. In this study, we did not review the captured image for whole specimens because it will be inconvenient in real practice. However, we reviewed the captured images of false negative result, but none of them could be edited to positive result.

We investigated the reasons for poor performance of the parameters in detecting 10^4^ CFU/ml bacteriuria. Applying the combination of WBC ≥6/HPF, ASP≥7500/μl, BACT≥1/HPF and positive LE, the false negative case numbers for detecting 10^5^ CFU/ml bacteriuria and 10^4^ CFU/ml bacteriuria were 21 and 61, respectively. This indicates that 65.6% (40/61) of false negative cases were from samples with 10^4–5^ CFU/ml bacterial concentration.

It is of note that, *Enterococcus* spp. (55.7%, 34/61) was the most common uropathogen causing the false negative results and was followed by *Escherichia coli* (23.0%,14/61), while the most common uropathogen detected was *Escherichia coli* (40.9%) and was followed by *Enterococcus* spp. (36.8%). In other words, as many as 31.8% (34/107) of *Enterococcus* spp.-grown specimens showed false-negative results, whereas only 11.8% (14/119) of *E. coli*- grown specimens showed false-negative results. This is in line with previous reports [10, 14], where images of iQ200 module were difficult to interpret as “bacteria”, except rod-form bacteria. In addition, *E. coli* was the most common pathogens among the 10^5^ CFU/ml bacteriuria, while *Enterococcus* spp. was the most common pathogen among the 10^4–5^ CFU/ml bacteriuria. This might be the reason why the performance of the parameters were lower in detecting 10^4^ CFU/ml compared to 10^5^ CFU/ml. In addition, in this study, growth of *Enterococcus* spp. accounted for as many as 36.8% of total culture-positive specimens, which is much higher than those of other studies (8–20%) [6, 7, 15]. Most cases of *Enterococcus* spp. were isolated from patients with hematologic malignancy (about 60–70%). As our hospital has the largest bone marrow transplantation center in Asia, the high proportion of specimens from the patients with hematologic malignancy might have influenced on the relatively lower sensitivity of this study than other study [6, 7].

In detecting WBC among the culture-positive cases, the positive rate of iQ200 was significantly higher than that of Gram stain. This finding is supported by previous studies comparing the iQ200/iChem workstation with the conventional method where iQ200/iChem workstation showed good correlation in WBC count [10, 11, 14]. Considering that pyuria is an important marker for UTI, detection of WBC by iQ200 will be useful.

Another important issue is the categorical approach for patient population. In this study, the sensitivity of two parameters (WBC and ASP) was less sensitive for specimens from adult women, especially elderly, than that for adult men. One previous study using Symex UF-500i flowcytometer [16] also found that bacteria and leukocyte count performed better in men and adult than in women and elderly. This is probably due to the high prevalence of UTI in women and elderly. Although urine culture is not necessary for outpatient with uncomplicated UTIs, it is mandatory for specific populations including elderly, pregnant women, infants, patient with spinal cord injuries, diabetes, underlying urologic abnormalities, immune compromised condition and indwelling urethral catheters [17].

## Conclusion

Based on our results, iQ200/iChem showed high NPV and culture reduction rate with the criteria of 10^5^ CFU/ml bacteriuria and in detecting pyuria which is the important marker for UTI, the positive rate of pyuria was higher with iQ200 than Gram stain. However, it did not show high enough performance for detecting bacteriuria in concentration of 10^4-5^ CFU/ml, mostly due to *Enterococcus* species. Taken together, iQ200/iChem workstation can be used for screening of urine specimens to reduce bacterial culture with the exception of specimens from the specific populations mentioned above.

## Additional file

Additional file 1: The raw dataset of 1942 urine samples including patients' gender, age-range, specimen type, and test results from culture, gram staining and iQ200/iChem workstation. (XLSX 251 kb)

Additional file 2: **Table S1.** Performance of combined parameters for detection of 10^5^ CFU/ml. (DOCX 28 kb)

## Data Availability

All data generated during the current study are included in this published article and its supplementary information file (Additional file 1).

## Abbreviations

ASP: All Small Particles
BACT: Bacteria
BYST: Yeast
CFU: Colony Forming Units
LE: Leukocyte Esterase
NPV: Negative Predictive Value
NT: Nitrite
PPV: Positive Predictive Value
UTI: Urinary Tract Infection
WBC: White Blood Cells
