# Supplementary material for: Clinical usefulness of iQ200/iChem Velocity workstation for screening of urine culture
Source: BMC Infect Dis. 2019 Jun 17;19:531. doi: 10.1186/s12879-019-4141-x (PMC6580579; doi:10.1186/s12879-019-4141-x)
Supplement: Supplementary file 2 — Table S1. Performance of combined parameters for detection of 105 CFU/ml. (DOCX 28 kb) [file 12879_2019_4141_MOESM2_ESM.docx]

Appendix Table S-1. Performance of combined parameters for detection of 10^5^ CFU/ml .

| **Combined parameters^*^** | **SEN** | **SPE** | **PPV** | **NPV** | **culture reduction** |
| --- | --- | --- | --- | --- | --- |
| (WBC≥6) + (ASP≥9500) ^†^ | 92.3% | 52.4% | 22.1% | 97.9% | 53.0% |
| (WBC≥6) + (ASP≥8500) ^†^ | 91.5% | 52.0% | 21.8% | 97.7% | 52.8% |
| (WBC≥6) + (ASP≥7500) ^†^ | 91.5% | 51.5% | 21.7% | 97.7% | 52.3% |
| (WBC≥6) + (ASP≥5500) ^†^ | 91.9% | 50.6% | 21.4% | 97.7% | 51.4% |
| (WBC≥6) + (ASP≥3500) | 92.3% | 47.1% | 20.4% | 97.7% | 47.8% |
| (WBC≥6) + (ASP≥2500) | 92.7% | 43.0% | 19.2% | 97.6% | 43.7% |
| (WBC≥6) + (ASP≥2000) | 93.1% | 40.7% | 18.7% | 97.6% | 41.4% |
| (WBC≥6) + (ASP≥1700) | 93.1% | 37.8% | 18.0% | 97.4% | 38.4% |
| (WBC≥6) + (ASP≥1500) | 93.5% | 35.6% | 17.5% | 97.4% | 36.2% |
| (WBC≥6) + (ASP≥1200) | 94.8% | 31.4% | 16.8% | 97.6% | 31.9% |
| (WBC≥4) + (ASP≥9500) ^†^ | 91.9% | 49.8% | 21.1% | 97.7% | 50.5% |
| (WBC≥4) + (ASP≥8500) ^†^ | 91.9% | 49.6% | 21.1% | 97.7% | 50.4% |
| (WBC≥4) + (ASP≥7500) | 91.9% | 49.2% | 20.9% | 97.7% | 49.9% |
| (WBC≥4) + (ASP≥5500) | 92.3% | 48.3% | 20.7% | 97.7% | 49.0% |
| (WBC≥4) + (ASP≥3500) | 92.7% | 45.1% | 19.8% | 97.7% | 45.8% |
| (WBC≥4) + (ASP≥2500) | 93.1% | 41.6% | 18.9% | 97.6% | 42.2% |
| (WBC≥4) + (ASP≥2000) | 93.5% | 39.4% | 18.4% | 97.7% | 40.0% |
| (WBC≥4) + (ASP≥1700) | 93.5% | 36.6% | 17.8% | 97.5% | 37.2% |
| (WBC≥4) + (ASP≥1500) | 94.0% | 34.5% | 17.3% | 97.5% | 35.0% |
| (WBC≥4) + (ASP≥1200) | 95.2% | 30.4% | 16.7% | 97.7% | 30.8% |
| (WBC≥2) + (ASP≥9500) | 94.4% | 38.8% | 18.4% | 97.9% | 39.3% |
| (WBC≥2) + (ASP≥8500) | 94.4% | 38.8% | 18.4% | 97.9% | 39.3% |
| (WBC≥2) + (ASP≥7500) | 94.4% | 38.6% | 18.4% | 97.9% | 39.1% |
| (WBC≥2) + (ASP≥5500) | 94.4% | 38.2% | 18.3% | 97.9% | 38.7% |
| (WBC≥2) + (ASP≥3500) | 94.8% | 36.7% | 18.0% | 98.0% | 37.2% |
| (WBC≥2) + (ASP≥2500) | 95.2% | 34.7% | 17.6% | 98.0% | 35.1% |
| (WBC≥2) + (ASP≥2000) | 95.6% | 33.2% | 17.3% | 98.1% | 33.6% |
| (WBC≥2) + (ASP≥1700) | 95.6% | 31.6% | 17.0% | 98.0% | 31.9% |
| (WBC≥2) + (ASP≥1500) | 95.6% | 30.0% | 16.7% | 97.9% | 30.4% |
| (WBC≥2) + (ASP≥1200) | 96.4% | 26.7% | 16.1% | 98.1% | 27.0% |
| (WBC≥1) + (ASP≥9500) | 97.6% | 17.9% | 14.8% | 98.1% | 18.1% |
| (WBC≥1) + (ASP≥8500) | 97.6% | 17.8% | 14.8% | 98.1% | 18.0% |
| (WBC≥1) + (ASP≥7500) | 97.6% | 17.8% | 14.8% | 98.1% | 18.0% |
| (WBC≥1) + (ASP≥5500) | 97.6% | 17.8% | 14.8% | 98.0% | 18.0% |
| (WBC≥1) + (ASP≥3500) | 98.0% | 17.6% | 14.8% | 98.3% | 17.7% |
| (WBC≥1) + (ASP≥2500) | 98.4% | 17.1% | 14.8% | 98.6% | 17.2% |
| (WBC≥1) + (ASP≥2000) | 98.4% | 16.6% | 14.7% | 98.6% | 16.7% |
| (WBC≥1) + (ASP≥1700) | 98.4% | 16.3% | 14.7% | 98.6% | 16.4% |
| (WBC≥1) + (ASP≥1500) | 98.4% | 15.8% | 14.6% | 98.5% | 15.9% |
| (WBC≥1) + (ASP≥1200) | 98.4% | 14.6% | 14.4% | 98.4% | 14.7% |

^*^The combination of WBC and ASP is presented along with BACT≥1/HPF and positive LE.

^†^The combinations of cut-off values, which satisfy the acceptable value; the NPV ≥97% and culture reduction rate ≥50%.

Appendix Table S-1. Performance of combined parameters for detection of 10^4^ CFU/ml .

| **Combined parameters^*^** | **SEN** | **SPE** | **PPV** | **NPV** | **culture reduction** |
| --- | --- | --- | --- | --- | --- |
| (WBC≥6) + (ASP≥9500) | 82.4% | 53.2% | 28.4% | 93.0% | 53.0% |
| (WBC≥6) + (ASP≥8500) | 82.4% | 52.9% | 28.3% | 93.0% | 52.8% |
| (WBC≥6) + (ASP≥7500) | 82.9% | 52.6% | 28.2% | 93.2% | 52.3% |
| (WBC≥6) + (ASP≥5500) | 83.5% | 51.7% | 28.0% | 93.3% | 51.4% |
| (WBC≥6) + (ASP≥3500) | 85.2% | 48.2% | 27.0% | 93.5% | 47.8% |
| (WBC≥6) + (ASP≥2500) | 86.0% | 44.0% | 25.7% | 93.3% | 43.7% |
| (WBC≥6) + (ASP≥2000) | 87.1% | 41.7% | 25.2% | 93.5% | 41.4% |
| (WBC≥6) + (ASP≥1700) | 88.2% | 38.8% | 24.5% | 93.6% | 38.4% |
| (WBC≥6) + (ASP≥1500) | 89.1% | 36.6% | 24.0% | 93.7% | 36.2% |
| (WBC≥6) + (ASP≥1200) | 91.0% | 32.4% | 23.3% | 94.1% | 31.9% |
| (WBC≥4) + (ASP≥9500) | 82.9% | 50.6% | 27.4% | 92.9% | 50.5% |
| (WBC≥4) + (ASP≥8500) | 82.9% | 50.5% | 27.4% | 92.9% | 50.4% |
| (WBC≥4) + (ASP≥7500) | 83.5% | 50.1% | 27.4% | 93.1% | 49.9% |
| (WBC≥4) + (ASP≥5500) | 84.0% | 49.2% | 27.1% | 93.2% | 49.0% |
| (WBC≥4) + (ASP≥3500) | 85.7% | 46.1% | 26.4% | 93.5% | 45.8% |
| (WBC≥4) + (ASP≥2500) | 86.6% | 42.5% | 25.3% | 93.3% | 42.2% |
| (WBC≥4) + (ASP≥2000) | 87.4% | 40.3% | 24.8% | 93.4% | 40.0% |
| (WBC≥4) + (ASP≥1700) | 88.5% | 37.5% | 24.2% | 93.6% | 37.2% |
| (WBC≥4) + (ASP≥1500) | 89.4% | 35.4% | 23.8% | 93.7% | 35.0% |
| (WBC≥4) + (ASP≥1200) | 91.3% | 31.3% | 23.0% | 94.1% | 30.8% |
| (WBC≥2) + (ASP≥9500) | 87.1% | 39.5% | 24.5% | 93.2% | 39.3% |
| (WBC≥2) + (ASP≥8500) | 87.1% | 39.4% | 24.5% | 93.1% | 39.3% |
| (WBC≥2) + (ASP≥7500) | 87.4% | 39.3% | 24.5% | 93.3% | 39.1% |
| (WBC≥2) + (ASP≥5500) | 87.4% | 38.9% | 24.4% | 93.2% | 38.7% |
| (WBC≥2) + (ASP≥3500) | 88.5% | 37.5% | 24.2% | 93.5% | 37.2% |
| (WBC≥2) + (ASP≥2500) | 89.4% | 35.5% | 23.8% | 93.7% | 35.1% |
| (WBC≥2) + (ASP≥2000) | 90.2% | 34.0% | 23.5% | 93.9% | 33.6% |
| (WBC≥2) + (ASP≥1700) | 91.0% | 32.4% | 23.3% | 94.1% | 31.9% |
| (WBC≥2) + (ASP≥1500) | 91.6% | 30.9% | 23.0% | 94.2% | 30.4% |
| (WBC≥2) + (ASP≥1200) | 93.0% | 27.6% | 22.4% | 94.6% | 27.0% |
| (WBC≥1) + (ASP≥9500) | 95.0% | 18.4% | 20.8% | 94.2% | 18.1% |
| (WBC≥1) + (ASP≥8500) | 95.0% | 18.3% | 20.7% | 94.2% | 18.0% |
| (WBC≥1) + (ASP≥7500) | 95.0% | 18.3% | 20.7% | 94.2% | 18.0% |
| (WBC≥1) + (ASP≥5500) | 95.0% | 18.2% | 20.7% | 94.1% | 18.0% |
| (WBC≥1) + (ASP≥3500) | 95.2% | 18.0% | 20.7% | 94.4% | 17.7% |
| (WBC≥1) + (ASP≥2500) | 95.8% | 17.6% | 20.8% | 94.9% | 17.2% |
| (WBC≥1) + (ASP≥2000) | 95.8% | 17.0% | 20.6% | 94.7% | 16.7% |
| (WBC≥1) + (ASP≥1700) | 95.8% | 16.7% | 20.6% | 94.6% | 16.4% |
| (WBC≥1) + (ASP≥1500) | 95.8% | 16.2% | 20.5% | 94.5% | 15.9% |
| (WBC≥1) + (ASP≥1200) | 96.4% | 15.0% | 20.3% | 94.8% | 14.7% |

^*^The combination of WBC and ASP is presented along with BACT≥1/HPF and positive LE.
